# Successful treatment of chylous leakage with delayed presentation after endometrial cancer surgery using dietary therapy, octreotide, and computed tomography‐guided lymphangiography: A case report and literature review

**DOI:** 10.1111/jog.70066

**Published:** 2025-08-31

**Authors:** Takayuki Takahashi, Iori Kisu, Shunki Kiyokawa, Mayuka Anko, Haruko Ohno, Teppei Okamura, Nobumaru Hirao

**Affiliations:** ^1^ Department of Obstetrics and Gynecology Federation of National Public Service Personnel Mutual Aid Associations, Tachikawa Hospital Tokyo Japan; ^2^ Department of Obstetrics and Gynecology Keio University School of Medicine Tokyo Japan; ^3^ Department of Radiology Federation of National Public Service Personnel Mutual Aid Associations, Tachikawa Hospital Tokyo Japan

**Keywords:** chylous ascites, CT‐guided lymphangiography, delayed presentation, endometrial cancer, lymphadenectomy, octreotide therapy

## Abstract

**Objective:**

Chylous ascites (CA) is a rare yet clinically significant complication following gynecologic cancer surgery, with incidence rates of 0.17 % to 9%. We aimed to describe a case of CA with a delayed clinical presentation nearly 100 days postoperatively in a patient with advanced endometrial cancer and to review the management strategies.

**Methods:**

We retrospectively evaluated a 75‐year‐old patient who underwent radical hysterectomy, bilateral salpingo‐oophorectomy, extended lymphadenectomy (pelvic and para‐aortic), and partial omentectomy for stage IIIB endometrial cancer. Data collected included onset timing, ascitic fluid analysis, imaging findings, and treatment responses. Additionally, a narrative review identified 13 relevant studies discussing the onset, risk factors, diagnosis, and therapies for post‐operative CA in gynecologic oncology.

**Results:**

Although CA typically appears within 4 to 21 days, our patient developed CA at approximately post‐operative day 99. Diagnostic paracentesis confirmed triglyceride‐rich ascitic fluid, establishing the diagnosis of CA. Dietary modification (fasting followed by medium‐chain triglyceride diet), octreotide therapy, and computed tomography (CT)‐guided lymphangiography effectively controlled the chylous leakage without requiring surgery.

Conservative measures—low‐fat or medium‐chain triglyceride diets, total parenteral nutrition, and somatostatin analogs—are considered first‐line, while lymphangiography/embolization and eventual surgical ligation may be needed for refractory cases.

**Conclusions:**

This case illustrates that CA with a delayed clinical presentation can be successfully treated with a stepwise conservative approach comprising dietary therapy, octreotide, and CT‐guided lymphangiography, even when presenting more than 3 months postoperatively. Moreover, our patient remained free of disease recurrence at 1 year and 8 months postoperatively, underscoring that timely management of CA can avoid delays in adjuvant therapy.

## INTRODUCTION

Chylous ascites (CA) is an uncommon yet clinically significant complication following gynecologic malignancy surgeries, with reported incidence rates for all post‐operative lymphatic leaks ranging from 0.17% to 9%.^1^, ^2^, ^3^, ^4^, ^5^ Studies that separately analyzed chylous ascites report a narrower range of 0.3% to 4.3%.^1^, ^2^, ^4^, ^9^ The extensive distribution of lymphatic vessels in the mesentery and retroperitoneum makes them susceptible to injury or obstruction during radical oncologic procedures.^6^ Such disruption can lead to electrolyte imbalances, immunodeficiency, and protein loss, with large‐volume ascites also causing discomfort, respiratory compromise, and malnutrition—all of which may delay adjuvant therapies.^7^, ^8^

Para‐aortic lymphadenectomy (PALND) has been identified as a prominent risk factor for post‐operative CA. Large‐scale studies consistently indicate that the extent of dissection correlates with CA onset, with a higher number of removed lymph nodes (LNs) increasing the risk^4^ (CA cohort),^9^ (mixed; CA subgroup ≈68%). For instance, an extensive retrospective analysis by Tulunay et al.^10^ demonstrated that the median number of para‐aortic LNs removed was significantly higher in patients who developed chylous ascites compared to those who did not (26 vs. 17, *p* = 0.001). Although CA is strongly associated with para‐aortic injury, it may also arise from purely pelvic dissections if critical lymphatic trunks are severed, as has been documented in several case reports.^11^, ^12^

**FIGURE 1:**
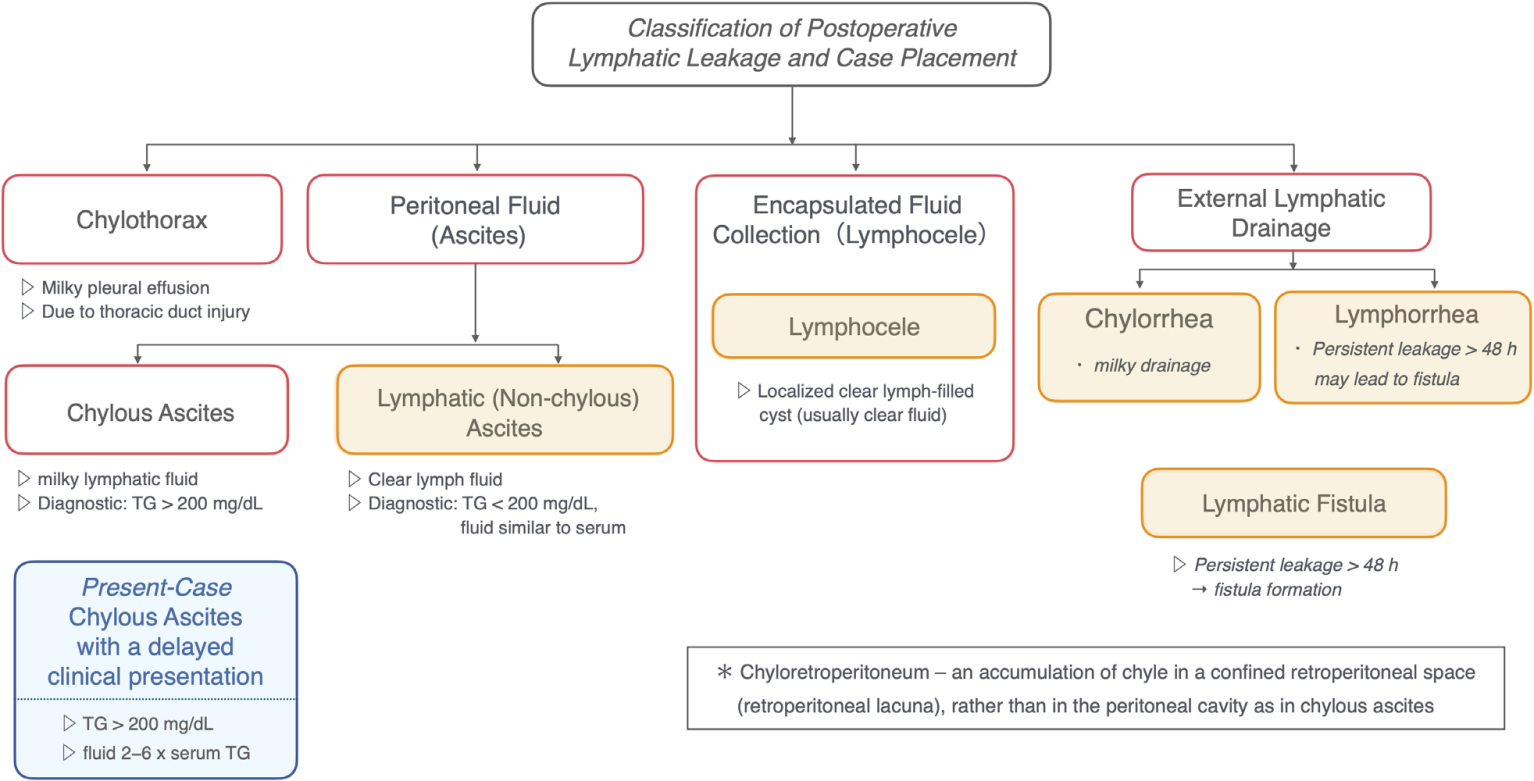
Classification of post‐operative lymphatic leakage and case placement. This review focuses exclusively on the entity outlined in the red frame—chylous ascites. This diagram categorizes lymphatic leaks by anatomical site (red frames) and fluid properties (orange frames). Thoracic duct injury leads to chylothorax (milky pleural effusion). In the peritoneal cavity, chylous ascites is milky fluid with TG >200 mg/dL, while lymphatic ascites is clear fluid with low TG. In soft‐tissue spaces, a lymphocele is an encapsulated collection of clear lymph. External wound leaks are chylorrhea (milky) or lymphorrhea (clear), which may progress to a lymphatic fistula if persistent beyond 48 h. Blue boxes highlight this case's chylous ascites with a delayed clinical presentation (TG >200 mg/dL) and conservative management (low‐fat/MCT diet ± drainage).

Post‐operative lymphatic leakage—manifesting as fluid accumulation in the abdominal cavity—can be broadly categorized as chylous ascites or lymphatic (non‐chylous) ascites.^13^, ^14^ Chylous ascites (chyloperitoneum) is a milky, triglyceride‐rich fluid originating from the intestinal lymphatics (e.g., thoracic duct, cisterna chyli). Typically caused by inadvertent surgical damage to these central lymphatic ducts, it is characteristically opalescent and has a triglyceride content often exceeding 200 mg/dL.^14^ By contrast, lymphatic ascites refers to a clearer, straw‐colored lymph leakage with a lower triglyceride concentration, usually stemming from smaller lymphatic channels that might be severed during nodal dissection.^14^ According to Lv et al., chylous ascites represents a distinct subtype of post‐operative lymphatic leakage. Figure 1 illustrates this classification, showing how our patient's condition aligns with “chylous ascites.”

Clinically, CA can appear within days to a few weeks postoperatively,^6^, ^15^ although delayed clinical presentation, beyond 70 days, has also been documented.^1^, ^7^ Such variability highlights the importance of long‐term surveillance after discharge; unrecognized CA with a delayed clinical presentation can severely disrupt patient care. Diagnosis hinges on ascitic fluid analysis demonstrating triglyceride >200 mg/dL,^8^, ^16^ often supported by imaging (e.g., ultrasound or computed tomography [CT]) to exclude alternative causes.^6^, ^16^

In many cases, conservative management proves effective. Dietary modifications, such as a low‐fat or medium‐chain triglyceride (MCT)‐enriched regimen, may be combined with paracentesis, total parenteral nutrition (TPN), or somatostatin analogs (e.g., octreotide) to reduce lymph flow and expedite leakage closure.^5^, ^6^, ^17^ A combination of low‐fat or MCT‐based diets and careful fluid management has decreased chylous leakage in the early post‐operative period.^18^ Additional evidence suggests that when supplemented by somatostatin analogs such as octreotide, dietary interventions expedite the closure of lymphatic leaks.^17^ Continuous subcutaneous octreotide may also facilitate the resolution of refractory CA by reducing lymph flow.^6^ Evidence also suggests that advanced interventional techniques—particularly CT‐guided lymphangiography—can localize and embolize the leakage site.^19^, ^20^ Minimally invasive procedures such as intranodal lymphangiography or embolization can avert repeated laparotomy in refractory cases.^21^, ^22^ If neither conservative nor interventional measures succeed, surgical options (e.g., peritoneovenous shunting or direct ligation) remain a final therapeutic option.^23^

Herein, we describe a case of CA with delayed clinical presentation following combined pelvic and para‐aortic lymphadenectomies for advanced endometrial cancer. Although CA is often attributed to para‐aortic injuries, our patient's leak originated in the pelvic region. We highlight how dietary therapy, octreotide administration, and CT‐guided lymphangiography resolved the leakage, obviating surgical re‐intervention. Finally, we compare our experience with the existing literature on CA risk factors, timing of onset, and management strategies, emphasizing the complexities of selecting an optimal treatment protocol.

## MATERIALS AND METHODS

### Study design and setting

This study comprised two main components: a detailed case report of a single patient who developed delayed clinical presentation of CA after extensive endometrial cancer surgery and a narrative literature review of previous studies reporting post‐operative CA in patients with gynecological malignancies. The patient was treated and followed at the Department of Obstetrics and Gynecology, Federation of National Public Service Personnel Mutual Aid Associations, Tachikawa Hospital, Tokyo, Japan. All diagnostic and therapeutic procedures were conducted in accordance with institutional guidelines. Informed consent was obtained from the patient for the publication of her clinical data. Our institutional review board does not require formal approval for single‐case reports or retrospective literature analyses.

### Case management

We retrospectively collected data on surgical findings, post‐operative course, timing of ascites onset, physical examination results, ascitic fluid analysis results, imaging examinations performed (CT and ultrasound), and laboratory test results. Responses to dietary modifications, subcutaneous octreotide treatment, and CT‐guided lymphangiography were documented.

Surgical details: The operative records were reviewed to assess key parameters (e.g., operative time, estimated blood loss, and extent of pelvic dissection and PALND).

Follow‐up strategy: During hospitalization, the patient's nutritional status, ascitic fluid output, imaging findings, and laboratory values were reviewed based on medical records. After discharge, records from regular outpatient visits were reviewed to assess for recurrence of ascites or other complications and to evaluate the patient's readiness to resume chemotherapy.

### Literature review

#### Search strategy

A literature search was performed in PubMed as part of a narrative review to identify studies reporting CA or lymphatic ascites after gynecologic oncology surgeries. The following search strategy was used:

((“chylous ascites” [MeSH Terms] OR “chylous ascites” OR “chyloperitoneum” OR “lymphatic ascites”) AND (“post‐operative period” [MeSH Terms] OR “post‐operative complications” [MeSH Terms] OR “post‐operative complication” OR “post‐operative” OR “lymph node dissection” OR “lymphadenectomy” OR “pelvic lymphadenectomy” OR “para‐aortic lymphadenectomy”)) AND (“Gynecologic Surgical Procedures” [MeSH Terms] OR “gynecologic surgery” OR “gynecological surgery” OR “gynecologic malignancies” OR “gynecological malignancies” OR “ovarian neoplasms” [MeSH Terms] OR “cervical neoplasms” [MeSH Terms] OR “endometrial neoplasms” [MeSH Terms] OR “vulvar neoplasms” [MeSH Terms] OR “vaginal neoplasms” [MeSH Terms]).

No language restrictions were initially applied, and the search included all studies indexed up to the time of the analysis. Titles and abstracts were screened independently by two authors (Takayuki Takahashi and M.K.).

#### Inclusion and exclusion criteria

Diagnostic eligibility

- Only studies that unequivocally reported post‐operative chylous ascites were eligible. Chylous ascites was defined as either (i) visibly milky peritoneal fluid and triglyceride (TG) >200 mg/dL or (ii) clear documentation of “chylous” or “chyloperitoneum” in conjunction with an elevated ascitic TG level. Studies describing non‐chylous (serous/clear) lymphatic ascites, congenital chylous malformations, or ascites of unclear biochemical profile were excluded.

The following studies were included in the analysis:

- Case reports, retrospective or prospective studies, or narrative/systematic reviews that describe post‐operative CA after pelvic, para‐aortic, or combined lymphadenectomy for gynecologic malignancies.
- Articles providing relevant information on the timing of onset, diagnostic criteria, treatment strategies, or outcomes.
- Full‐text articles accessible through institutional subscriptions or open‐access platforms

The following studies were excluded:

- Studies that focused solely on incidence rates or provided a summary of broader post‐operative complications without specific data on CA management.
- Studies that reported the onset of CA following non‐gynecological procedures or in populations different from those with gynecologic cancer
- Redundant articles or conference abstracts lacking full‐text data

#### Data extraction

Full‐text articles meeting the inclusion criteria were retrieved, and the following data were extracted:

- Study design and sample size (case reports, retrospective cohorts, prospective studies, or reviews)
- Patient demographics and the type of gynecologic malignancy (e.g., endometrial, cervical, or ovarian)
- Surgical procedures, including details of LN dissection (pelvic vs. para‐aortic)
- Timing of CA onset (days post‐surgery)
- Diagnostic methods (e.g., paracentesis, triglyceride measurement, and imaging)
- Treatment strategies (e.g., dietary modifications, TPN, octreotide, lymphangiography/embolization, or surgical re‐intervention)
- Prognostic outcomes, including ascites resolution, recurrence rates, and the impact on adjuvant therapy

Data extraction was performed independently by two authors. Discrepancies were resolved by consensus with a third author.

#### Data analysis and synthesis

A qualitative synthesis was conducted to summarize key trends in the timing of onset, diagnostic approaches, and therapeutic management of post‐operative CA across the selected studies. Particular focus was placed on the following aspects:

1. Risk factors for CA, including the extent of lymphadenectomy (pelvic vs. para‐aortic), number of LNs removed, and surgical approach used (open vs. laparoscopic/robotic)
2. Treatment outcomes, focusing on the success rates of dietary therapy, somatostatin analogs, and interventional radiology procedures
3. Implications for adjuvant therapy, specifically delays in the administration of chemotherapy or radiotherapy

Comparisons were made between the findings of the present patient and data reported in the literature. Results from the literature review were then integrated into the Discussion section to contextualize the clinical decision‐making process and to highlight effective strategies for managing delayed CA.

## RESULTS

### Case presentation

#### Patient information

A 75‐year‐old female patient first presented to our hospital in December 2023 with abnormal genital bleeding. She had a medical history of Stage IIA (T2N0M0, AJCC 8th Edition) left‐sided breast cancer treated previously, as well as hypertension, dyslipidemia, osteoporosis, and cataracts. She experienced menopause at 51 years old and had three vaginal deliveries. At her first presentation, she weighed 49 kg, stood 153 cm tall, and had a BMI of 20.93.

#### Case details

An endometrial biopsy of the patient's uterine lesion revealed grade 3 endometrioid adenocarcinoma. Preoperative contrast‐enhanced CT showed no evidence of distant metastasis. Pelvic magnetic resonance imaging (MRI) indicated possible extension to surrounding tissues and the cervix. According to the 2023 International Federation of Gynecology and Obstetrics (FIGO) staging system,^24^ the tumor was clinically diagnosed as stage IIA endometrial cancer with cervical stromal invasion. Definitive surgical intervention was planned.

#### Surgical procedure

The patient underwent a radical hysterectomy, bilateral salpingo‐oophorectomy, pelvic lymphadenectomy, para‐aortic lymphadenectomy (extending to the renal veins), and a partial omentectomy. The operation lasted 10 h 33 min, with an estimated blood loss of 547 mL and no significant intraoperative complications. Because an extensive lymphadenectomy was performed, a closed‐suction abdominal drain was placed in the left lower abdomen to facilitate early detection of hemorrhage or chyle leakage. Lymphatic channels in the para‐aortic region were meticulously sealed using bipolar energy devices and secured with ligatures. Pathological assessment of the 72 pelvic and 23 para‐aortic lymph nodes revealed no nodal metastasis. According to the FIGO 2023 staging guidelines, the final pathological stage was pT3bN0M0 (stage IIIB).

#### Post‐operative course, adjuvant therapy, and delayed clinical presentation of ascites

On post‐operative day (POD) 2, the patient began a liquid diet, which was advanced to a semisolid meal (half‐portion porridge) on POD3 and a near‐regular diet (full‐portion porridge plus milk) by POD4. To assess for chylous leakage, we introduced high‐fat dairy products on POD5. The intraperitoneal drain initially yielded 360 mL of serosanguineous fluid on POD1, followed by 280 mL on POD2, 126 mL on POD3, 86 mL on POD4, and 42 mL of serosanguineous fluid without any milky admixture on POD5. As the output remained non‐chylous and declined steadily, the drain was removed on POD5.

**FIGURE 2:**
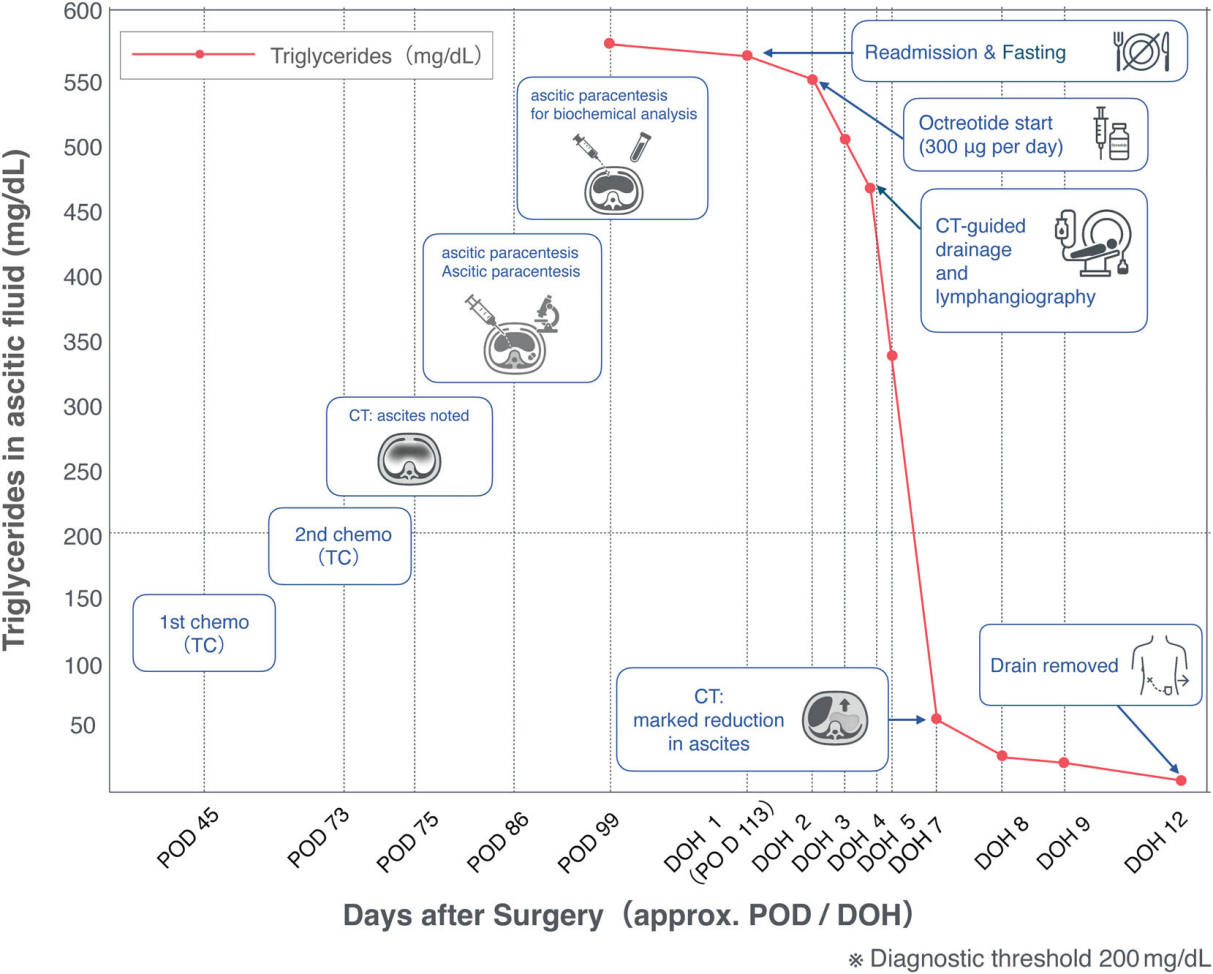
Timeline of the treatment and management of post‐operative chylous ascites and triglycerides in the ascitic fluid. This graph traces the patient's clinical course from the first chemotherapy cycle on post‐operative day (POD) 45 through hospital readmission, intervention, and subsequent resolution of chylous ascites. The x‐axis shows days after surgery (approximate POD or Day of Hospitalization [DOH]), and the y‐axis plots ascitic triglyceride (mg/dL). The first cycle of paclitaxel–carboplatin (TC) chemotherapy was administered on POD45, followed by a second cycle on POD73. A CT scan on POD75 revealed ascites. Despite a benign‐appearing effusion on POD86, delayed clinical presentation of chylous ascites was confirmed on POD99 (580 mg/dL). After readmission on POD113 (DOH1), ascitic fluid triglyceride was 567 mg/dL. Continuous subcutaneous octreotide (300 μg/day) began on POD114 (DOH2). Further paracenteses showed triglyceride levels of 552 mg/dL (DOH2), 465 mg/dL (DOH4), 52 mg/dL (DOH7), 26 mg/dL (DOH8), 23 mg/dL (DOH9), and 8 mg/dL (DOH12). Lymphangiography and CT‐guided drainage were performed on DOH4, after which the ascitic fluid output gradually decreased. By DOH12, the patient could tolerate a high‐fat diet without re‐accumulating chylous fluid, and the drain was removed. She was discharged home on DOH14 and resumed adjuvant chemotherapy without further complications. CT, computed tomography; DOH, Day of hospitalization (e.g., DOH1 = hospitalization day 1); POD, Post‐operative day; TC, Paclitaxel + Carboplatin.

**FIGURE 3:**
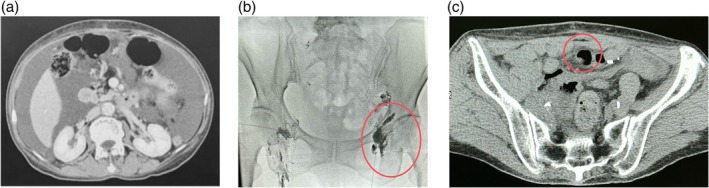
Imaging studies of chylous ascites detection and lymphatic leakage localization. (a) Abdominal CT on post‐operative day 75 showing significant accumulation of ascitic fluid in the pelvic cavity. (b) Although initial lymphangiography did not reveal any leakage, ultrasound‐guided puncture of the left inguinal lymph node with lipiodol injection confirmed intraperitoneal leakage from the left external iliac lymph node. (c) A CT scan (cross‐sectional view) after lipiodol injection into the left and right pelvic lymph nodes shows leakage of contrast medium into the abdominal cavity (leakage site marked in red), indicating lymphatic leakage originating from the left external iliac lymph nodes. The lymph fluid leakage is indicated in red.

The first paclitaxel–carboplatin (TC) chemotherapy cycle began on POD45, with a second cycle administered on POD73 (Figure 2). However, on POD75, a follow‐up CT scan revealed a substantial volume of new‐onset pelvic ascites (Figure 3a). Although malignant ascites were initially suspected, a transabdominal paracentesis on POD86 demonstrated fluid composed primarily of mesothelial cells, red blood cells, neutrophils, and lymphocytes—findings suggest a benign effusion. Around this time, the patient acknowledged increasing dietary fat intake at home, and by POD99, she had developed further abdominal distension. A repeat paracentesis on POD99 confirmed CA, with an ascitic fluid triglyceride level of 580 mg/dL, total cholesterol of 98 mg/dL, and a normal serum triglyceride of 106 mg/dL. Given these findings and the delayed post‐operative interval, the patient was admitted for inpatient management on POD113.

#### Inpatient management and clinical course

On POD 113, the patient was readmitted and designated as hospitalization day 1 (DOH1). At this time, paracentesis revealed an ascitic fluid triglyceride level of 567 mg/dL, while serum triglyceride remained normal at 88 mg/dL, confirming the chylous nature of the ascites. The patient was instructed to fast, and continuous subcutaneous octreotide (300 μg/day) was initiated on POD114 (DOH2), corresponding to DOH2. At that point, a follow‐up paracentesis still showed ascitic fluid triglycerides of 552 mg/dL.

On DOH4, lymphangiography under ultrasound guidance was performed by injecting Lipiodol into the left inguinal lymph node, which localized a chylous leak around the left external iliac lymph node region (Figure 3b,c). A 6Fr drainage catheter was placed in the pelvic cavity. Analysis of ascitic fluid on DOH4 demonstrated a triglyceride level of 465 mg/dL; serum triglyceride remained normal at 85 mg/dL. Daily measurements indicated a steady decline in ascitic fluid triglycerides: 52 mg/dL on DOH7, 26 mg/dL on DOH8, 23 mg/dL on DOH9, and 8 mg/dL on DOH12.

Following an initial fasting period, a low‐fat diet enriched with medium‐chain triglycerides (MCT) was gradually introduced to reduce lymphatic flow. By DOH12, the patient could tolerate a high‐fat challenge (including milk) without re‐accumulating chylous fluid, and the drainage catheter was removed. Imaging confirmed a marked reduction in ascites and stable serum triglyceride levels (82 mg/dL). The patient demonstrated significant clinical improvement and was discharged on DOH14. Adjuvant chemotherapy resumed promptly, and at the 1‐year and 8‐month follow‐up, she remained free of disease recurrence, suggesting a favorable long‐term prognosis.

### Literature review of 13 relevant studies

#### Overview of the 13 relevant studies

Our literature search and rigorous re‐evaluation identified 13 studies that met our strict inclusion criteria for post‐operative CA in gynecologic oncology. These studies, comprising case reports and small case series, provided the data for our narrative review. The key findings from these studies, including patient characteristics, management strategies, and outcomes, are summarized in Table 1.

**TABLE 1:** Summary of 13 relevant studies on postoperative chylous ascites in gynecologic malignancies.

Author (year)	Design	Cancer type/LN dissection	Diagnostic evidence	Onset/course of CA (POD = postoperative day)	Management	Outcome
Horibe (2024)	Case rpt (*n* = 1)	46F, OvCa IA + EndoCa; Pelvic + PA LN	Chylous ascites (TG not reported)	POD1, refractory; multiple LAGs failed to find leak	Fasting, low‐fat diet, repeated drainage, 2× Lipiodol LAG	Resolved by POD142; no recurrence
Tan (2024)	2 cases + review	Case1: 14F OvCa germ tumor; Case2: 55F ESS (no LN dissection)	Milky‐white fluid (TG ~393 & ~373 mg/dL)	Onset POD4 & POD9	Diet restriction (low‐fat/no‐fat) + TPN	Both resolved by POD15; no reoperation
GilGonzález (2022)	Case rpt (n = 1)	66F, CervCa IIB; Laparoscopic PA LN	“Bloody milky” fluid (TG 357 mg/dL)	~1 month post‐op	High‐protein, low‐fat, MCT diet, octreotide, paracentesis	Rapid improvement; no ascites at 1 month
Lee (2018)	Case rpt (letter)	73F, CervCa IIB; Pelvic + PA LN	Milky fluid (TG 683 mg/dL)	POD3, massive (>2 L/day); refractory to conservative mgt	Mesenteric LN intranodal lipiodol injection	Resolved in 3 days; no recurrence (14 mo)
GoiriLittle (2014)	Case rpt (n = 1)	61F, EndoCa (>50% invasion); Laparoscopic Pelvic + PA LN	Milky fluid (TG not reported)	POD22 (developed over 3 days)	IV somatostatin, low‐fat/MCT diet, paracentesis	Resolved by ~POD30; no recurrence
Kim EA (2014)	Case rpt (n = 1)	43F, EndoCa; Laparoscopic Pelvic + PA LN	White milky fluid (TG not reported)	POD4 (~2000 mL/day); refractory to diet/TPN for 40 days	Added subcutaneous octreotide	Fully resolved; no recurrence (14 mo)
Shibuya (2013)	Case rpt (n = 1)	60F, EndoCa Stage II; Pelvic LN only	Milky fluid (TG 677 mg/dL)	POD41; refractory to paracentesis	Low‐fat/MCT diet + continuous low‐pressure drainage (9 days)	Resolved by POD60; no recurrence
Soto (2011)	Case rpt (n = 1)	38F, CervCa IB2/IIIB; Robotic Pelvic + PA LN	Milky‐fluid (TG 236 mg/dL)	POD9	TPN, low‐fat/MCT diet, octreotide, paracentesis	Resolved in 6 weeks; no recurrence (40 mo)
Baiocchi (2010)	2 case rpts + review	(1) 50F, OvCa IIIC; (2) 63F, EndoCa IBG3; Both Pelvic + PA LN	Milk whitish fluid (TG 482 & 350 mg/dL)	Case1: POD9; Case2: POD4	TPN, low‐fat/MCT diet, octreotide, drainage	Both resolved; no recurrence of CA
Dewdney (2006)	Case rpt (n = 1)	19F, history of OvCa immature teratoma; Resection of retroperitoneal mature teratoma	Initial ascites clear on POD22; became chylous (cloudy, TG 305 mg/dL) on POD39	TPN, low‐fat/MCT diet, multiple paracenteses	Spontaneously resolved after ~11 weeks	Spontaneously resolved after ~3 months
Takeuchi (2006)	Case rpt (n = 1)	56F, CervCa IIIB; Pelvic + PA LN	Milky white fluid (TG not reported)	POD4 (~1800 mL/day), continued on POD5	Fasting, TPN, low‐fat diet, drainage	Resolved by POD24; no surgery
Boran (2004)	Case rpt (n = 1)	35F, Borderline mucinous ovarian tumor; Pelvic + PA LN	Milky fluid (TG 421 mg/dL)	POD11; refractory to conservative mgt	Surgical ligation of fistula on cisterna chyli	Resolved after surgery; no recurrence (10 mo)
Manolitsas (2002)	Case rpt (*n* = 2)	(1) 66F, CervCa IIB; (2) 78F, Uterine MMMT; Both Pelvic + PA LN	Milky white fluid (Case 2: TG 1032 mg/dL)	Case1: ~10mo post‐RT; Case2: <10d post‐PALND	Paracentesis, TPN/low‐fat diet, Denver shunt	Both improved after shunt placement

Abbreviations: CA, Chylous ascites; CervCa, Cervical cancer; EndoCa, Endometrial cancer; ESS, Endometrial stromal sarcoma; F, Female; IV, Intravenous; LAG, Lymphangiography; LN, Lymph node; mgt, Management; MMMT, Malignant mixed Müllerian tumor; mo, Month(s); MCT, Medium‐chain triglyceride; OvCa, Ovarian cancer; PA, Para‐aortic; POD, Postoperative day; rpt, Report; RT, Radiotherapy; TG, Triglyceride; TPN, Total parenteral nutrition.

#### Onset timing

The timing of the clinical presentation of CA varied widely across the reviewed studies, from as early as the first post‐operative day to as late as approximately 1‐month post‐surgery.^25^, ^26^ Several cases were diagnosed within the first 2 weeks, with specific onsets reported on post‐operative day 3,^22^ day 4,^6^, ^16^, ^27^, ^28^ and day 9.^27^, ^29^ Other cases presented later, at day 11,^15^ day 22,^30^ and day 41.^12^ One unique case was reported where ascites was initially clear on day 22 and became clinically chylous on day 39.^31^

#### Diagnostic approaches

The diagnosis of CA in all reviewed cases was primarily established through paracentesis or analysis of fluid from a surgical drain. A milky or chylous appearance of the fluid was a consistent finding across most reports.^6^, ^12^, ^15^, ^23^, ^28^, ^30^ In many cases, this clinical finding was supported by objective biochemical analysis demonstrating elevated ascitic fluid TG levels, with reported values including 236 mg/dL,^29^ >350 mg/dL,^27^ 357 mg/dL,^26^ 421 mg/dL,^15^ 677 mg/dL,^12^ 683 mg/dL,^22^ and 1032 mg/dL.^23^ In some cases, while the fluid was described as chylous, TG levels were not reported.^25^ For refractory cases where the leakage site could not be identified, advanced imaging such as lymphangiography was utilized.^22^, ^25^

#### Management strategies and outcomes

Most published cases initially employed conservative measures, typically including dietary modifications such as a low‐fat diet with medium‐chain triglycerides (MCT) and/or total parenteral nutrition (TPN), often combined with somatostatin analogs like octreotide or somatostatin.^6^, ^16^, ^26^, ^27^, ^28^, ^29^, ^30^, ^31^ One study reported the use of a novel continuous low‐pressure drainage system.^12^

Most patients responded successfully to conservative management, with reported resolution times ranging from a few days to several weeks.^16^, ^29^ However, some cases proved refractory to these initial measures. In one such case, a mesenteric intranodal lymphangiography with Lipiodol injection was successful.^22^ For cases that failed all non‐surgical options, invasive procedures were required. These included surgical ligation of the leaking fistula^15^ and the placement of a peritoneovenous (Denver) shunt.^23^ One study described a case of spontaneous resolution after multiple failed lymphangiographies and a period of outpatient standby therapy.^25^

## DISCUSSION

### Significance of delayed clinical presentation of chylous ascites

Post‐operative lymphatic leakages are heterogeneous, but CA is readily recognized by its opalescent, triglyceride‐rich fluid (usually >200 mg/dL) that originates from disruption of intestinal lymphatic ducts, cisterna chyli, or thoracic duct.^14^ Although uncommon, CA is a clinically significant after gynecologic cancer surgery, with reported incidence rates for all postoperative lymphatic leaks ranging from 0.17% to 9%^1^, ^4^, ^5^, ^9^; studies that separately analyzed CA report a narrower range of 0.3%–4.3%.^1^, ^2^, ^4^, ^9^

As summarized in Section 3.2.2, most postoperative chylous ascites cases manifest clinically within the first 2–3 weeks^16^, ^29^; therefore, our patient's presentation beyond this window underscores the rarity of a truly delayed clinical presentation. However, several authors—including Manolitsas et al. and Horibe et al.—have described much later presentations, 70–140 days after surgery.^23^, ^25^ Such latency hampers recognition: by the time symptoms occur, patients have resumed a regular, higher‐fat diet, increasing gut lymph flow and converting a silent leak into overt ascites. Our patient followed this pattern, developing tense ascites on post‐operative day approximately 100 shortly after increasing dietary fat at home. These observations emphasize the need for prolonged surveillance, including clinical review, patient education about diet, and a low threshold for imaging or diagnostic paracentesis, for at least 3 months after extensive para‐aortic or pelvic lymphadenectomy.

Large‐volume chyle loss causes protein–calorie malnutrition, lymphopenia, electrolyte imbalance, and an increased risk of sepsis, each of which can compromise tolerance to adjuvant chemotherapy. Prompt diagnosis enables the early institution of dietary fat restriction, somatostatin analogues, and, when indicated, image‐guided lymphatic interventions, thereby preserving nutritional status and preventing treatment delays.

### Risk factors and pathophysiology

The literature consistently identifies the breadth of retroperitoneal lymph‐node dissection—especially the removal of para‐aortic nodes—as the dominant surgical determinant of post‐operative CA.^1^, ^4^, ^9^ In the largest retrospective cohorts to date, the probability of CA rises in parallel with the number of nodes excised; Solmaz and colleagues found a stepwise increase once more than 14 para‐aortic nodes were removed.^9^ Although most leaks originate where central intestinal lymphatics join the cisterna chyli or thoracic duct, clinical experience shows that an isolated pelvic dissection can also be sufficient if a critical efferent trunk is divided, as illustrated by Shibuya's case of high‐volume, triglyceride‐rich ascites (TG 677 mg/dL) after pelvic lymphadenectomy alone.^12^

**FIGURE 4:**
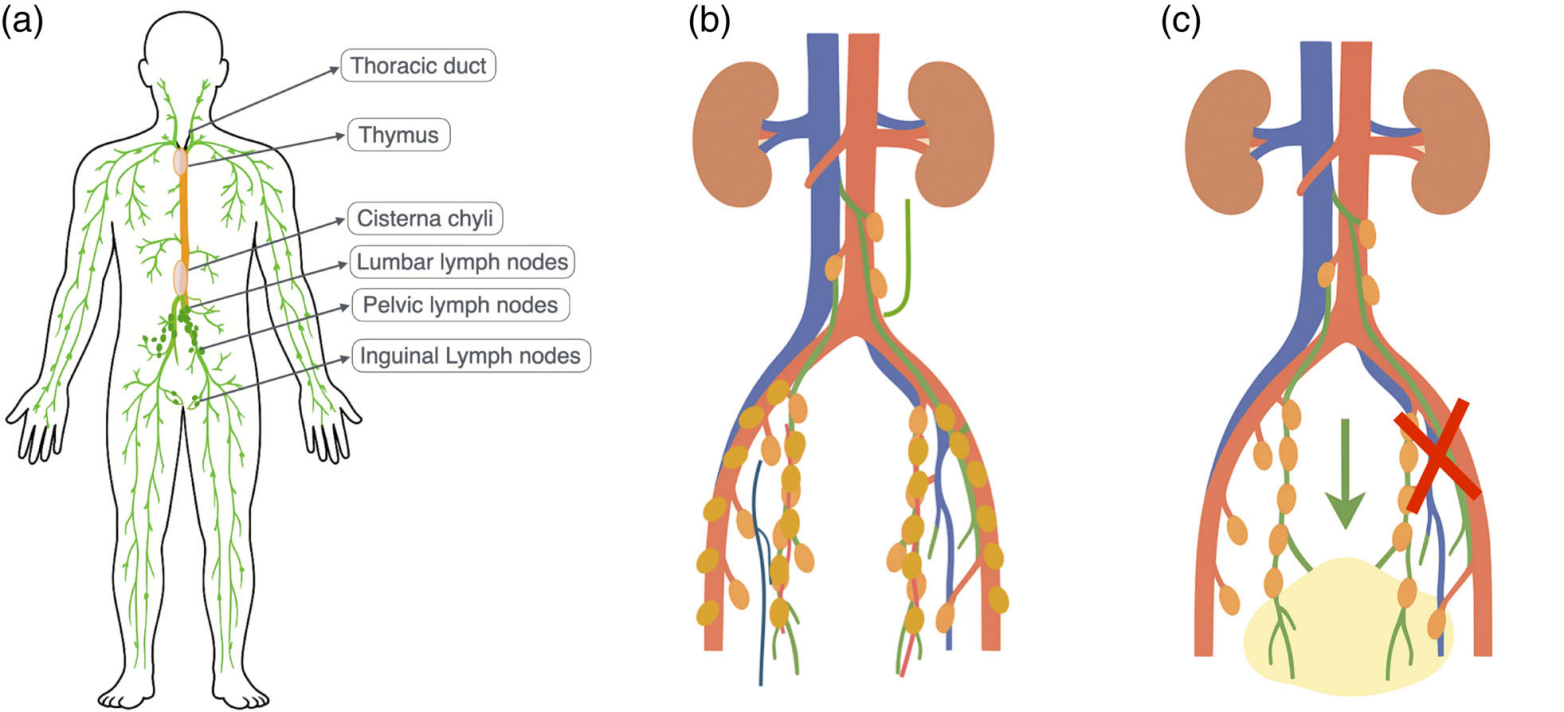
Schematic illustration of the lymphatic system and the pathogenesis of chylous ascites in the present case. (a) Whole‐body lymphatic circulation, showing how lymph (chyle) from the lower body and gastrointestinal tract collects in the cisterna chyli and ascends via the thoracic duct to drain into the venous system. (b) Detailed anatomy of the para‐aortic and pelvic lymph nodes and lymphatic channels relevant to gynecologic surgery. (c) Proposed mechanism of chylous ascites in this case. Extensive dissection and disruption of high‐level para‐aortic lymphatic channels obstruct the normal cranial flow of lymph. This obstruction may lead to increased pressure and retrograde (cranial‐to‐caudal) flow (indicated by arrow) down remaining collateral channels. The elevated pressure can then cause a rupture of a smaller lymphatic vessel at a more distal site, such as the left external iliac chain identified in our patient, resulting in chylous ascites. In this figure legend, panel (a) provides context by depicting normal lymphatic drainage from the abdomen and pelvis. Panel (b) focuses on the lymph node groups (para‐aortic, pelvic, external iliac, etc.) typically involved in gynecologic cancer surgery. Panel (c) illustrates the post‐operative lymphatic leakage specific to the case, highlighting the site of disruption and the flow of chyle into the abdominal cavity. This comprehensive caption ensures the figure is self‐explanatory and clearly tied to the case discussion.

The location of the chylous leak in the external iliac region, despite an extensive para‐aortic dissection extending to the renal veins, presents a complex pathophysiological question. One plausible mechanism is retrograde lymphatic flow, as illustrated in Figure 4. The high‐level disruption and obstruction of the primary cephalad lymphatic pathways could have led to increased pressure within the lymphatic system, causing chyle to flow backward from the cisterna chyli down remaining collateral channels into the pelvis. This elevated pressure may have resulted in a rupture of a smaller lymphatic vessel in the external iliac chain. Moreover, we cannot exclude the possibility that a second, undetected leak was present at a more cranial level, allowing chyle to track downward and collect in the pelvis.

### Management strategies: A stepwise and individualized approach

**FIGURE 5:**
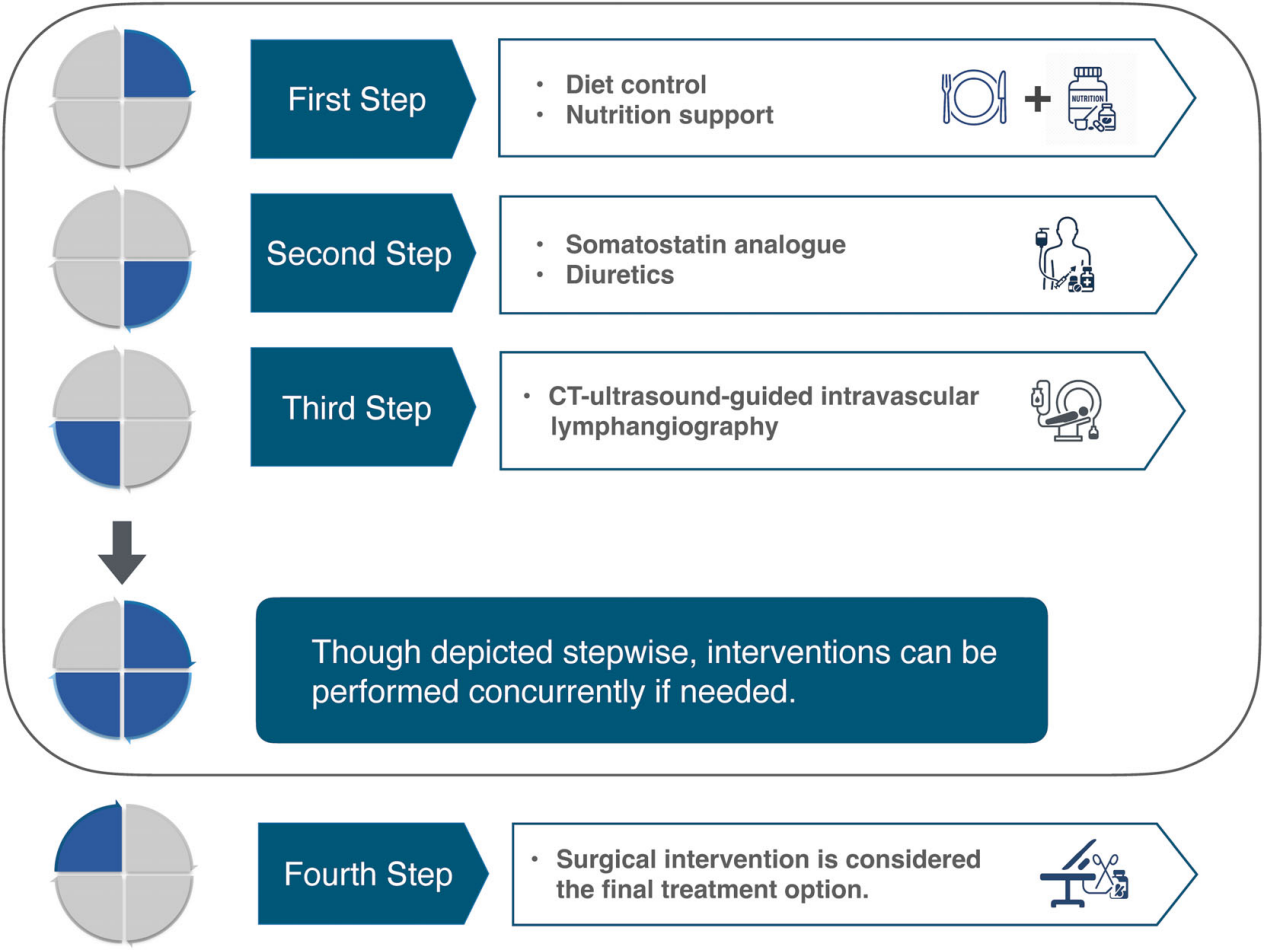
Proposed stepwise algorithm for managing post‐operative chylous ascites. First‐line treatment is conservative, involving dietary modification and somatostatin analogs. Interventional radiology (e.g., lymphangiography and embolization) is recommended if there is no improvement after 7 to 10 days, or earlier if “early‐escalation” criteria are met. These criteria include high‐output drainage (>500 mL/day), rapid reaccumulation of ascites, or persistently high ascitic triglyceride levels. Surgical intervention is reserved for cases that are refractory to other treatments.

Based on our experience and the literature review, we propose a stepwise algorithm for managing CA (Figure 5). First‐line treatment involves conservative therapy, including dietary modification (such as fasting or a low‐fat, medium‐chain triglyceride‐enriched diet) and somatostatin analogs like octreotide. If improvement is not observed within 7 to 10 days, or earlier when any of the following “early‐escalation” criteria are met—(i) high‐output drainage >500 mL/day, (ii) rapid re‐accumulation after repeated paracentesis, or (iii) persistently elevated ascitic triglycerides >400 to 500 mg/dL—image‐guided lymphangiography with possible embolization is recommended. Surgery, such as direct ligation or peritoneovenous shunting, remains the final option for refractory cases.

Consequently, when ascites persists beyond 7 to 10 days or fulfills any early escalation criterion described above despite conservative therapy, the probability of spontaneous closure diminishes, warranting prompt interventional radiology. Several studies support this staged approach. Although CA‐specific data on spontaneous closure are scarce, limited evidence from non‐chylous lymphatic ascites may offer a pragmatic reference for the initial observation window. In a small series of non‐chylous post‐operative lymphatic ascites in endometrial cancer, Takefushi et al. reported a median spontaneous closure time of 11 days. While pathophysiological differences exist, this finding supports using a 1 to 2 week observation window before escalation in presumed CA.^13^ Additionally, Tan et al. reviewed 140 gynecologic cases of post‐operative chylous ascites, reporting a 92.86% success rate using only conservative management, with a median resolution time of 9 days.^16^ These findings suggest that in most cases, 1 to 2 weeks of dietary restriction, TPN, and/or somatostatin analogs can achieve closure.

Indeed, a multicenter meta‐analysis by Rose et al. showed that lipiodol lymphangiography and embolization successfully resolved chylous ascites in up to 97% of refractory cases following initial conservative failure, with the remaining few patients managed by surgical ligation or shunting.^32^

This stepwise escalation can be adapted to individual patient presentations, as demonstrated in our case. The management of our patient aligns with this refined algorithm; after initiating conservative therapy, the patient continued to have high‐output drainage exceeding 1 L/day with persistently elevated triglyceride levels. Thus, meeting the criteria we proposed for early intervention, a CT‐guided lymphangiography was appropriately performed on day 3 of admission. This application of our tiered approach was followed by a rapid resolution of the CA, successfully avoiding the need for surgical intervention.

### Clinical significance of continuous drainage with serial TG monitoring

A notable aspect of our case management was the use of continuous drainage coupled with serial monitoring of ascitic TG levels. While prolonged drainage carries a risk of malnutrition (Reference 7), serial TG monitoring provided crucial, real‐time feedback on the effectiveness of our conservative measures. The rapid decline in TG levels following fasting and octreotide therapy confirmed the closure of the chylous fistula, even while serous fluid drainage continued (Figure 2). This biochemical evidence allowed us to confidently advance the patient's diet from fasting to a low‐fat MCT diet, and ultimately to challenge with a high‐fat diet, without fear of recrudescence. Therefore, we suggest that serial TG monitoring can be a valuable tool to precisely guide dietary advancement and determine the optimal timing for drain removal, thereby mitigating some of the nutritional risks associated with prolonged drainage. In our practice, we used ascitic TG <100 mg/dL as a practical management target indicating biochemical resolution to guide diet advancement and drain removal; this target is distinct from the diagnostic threshold for CA (ascitic TG >200 mg/dL).

### Limitations

First, this is a single‐case report, and causality between our TG‐guided decisions and the outcome cannot be proven. Second, the narrative review combined heterogeneous case series with inconsistent threshold values for TG; therefore, the pooled statistics were inappropriate. Third, lymphoscintigraphy was unavailable at our center; a second occult cranial leak cannot be excluded. Multicenter registries using uniform diagnostic definitions (ascitic TG >200 mg/dL to define CA) are needed to validate the proposed escalation triggers.

## CONCLUSION

Late‐presenting CA can be cured without surgery when a structured, TG‐guided algorithm is combined with octreotide and CT‐lymphangiography. Early escalation, informed by drainage rate and biochemical trends, prevents nutritional decline and preserves the timeline for adjuvant therapy. Multicenter data are warranted to test the proposed management cut‐off for biochemical resolution (ascitic TG <100 mg/dL) and to validate escalation triggers.

## AUTHOR CONTRIBUTIONS

**Takayuki Takahashi:** Conceptualization; data curation; formal analysis; investigation; methodology; project administration; resources; software; supervision; validation; visualization; writing – original draft; writing – review and editing. **Iori Kisu:** Funding acquisition; methodology; supervision; writing – review and editing. **Shunki Kiyokawa:** Software; writing – original draft; writing – review and editing. **Mayuka Anko:** Writing – review and editing. **Haruko Ohno:** Writing – review and editing. **Teppei Okamura:** Writing – review and editing. **Nobumaru Hirao:** Writing – review and editing.

## FUNDING INFORMATION

The authors received no financial support for the research, authorship, or publication of this article.

## CONFLICT OF INTEREST STATEMENT

The authors declare that they have no known competing financial interests or personal relationships that could have appeared to influence the work reported in this article.

## ETHICS STATEMENT

Our institution does not require ethical approval to report individual case reports or case series.

## INFORMED CONSENT

Informed consent was obtained from the patient for the publication of patient information.

## Data Availability

The data underlying this case report are based on individual patient medical records. To protect patient privacy and in accordance with institutional ethical policies, these data are not publicly available. De‐identified data are available from the corresponding author upon reasonable request.
